# Flavin-containing monooxygenase 3 (FMO3) role in busulphan metabolic pathway

**DOI:** 10.1371/journal.pone.0187294

**Published:** 2017-11-09

**Authors:** Ibrahim El-Serafi, Ylva Terelius, Manuchehr Abedi-Valugerdi, Seán Naughton, Maryam Saghafian, Ali Moshfegh, Jonas Mattsson, Zuzana Potácová, Moustapha Hassan

**Affiliations:** 1 Experimental Cancer Medicine (ECM), Clinical Research Centre (KFC), Department of Laboratory Medicine, Karolinska Institutet, Huddinge, Stockholm, Sweden; 2 Cancer Center of Karolinska (CCK), Department of Oncology-Pathology, Karolinska Institutet, Solna, Stockholm, Sweden; 3 Centre for Allogeneic Stem Cell Transplantation, Karolinska University Hospital-Huddinge, Stockholm, Sweden; 4 Department of Oncology and Pathology, Karolinska Institutet, Solna, Stockholm, Sweden; 5 ECM, Clinical Research Centre (KFC), Novum, Karolinska University Hospital, Huddinge, Sweden; University of Insubria, ITALY

## Abstract

Busulphan (Bu) is an alkylating agent used in the conditioning regimen prior to hematopoietic stem cell transplantation (HSCT). Bu is extensively metabolized in the liver via conjugations with glutathione to form the intermediate metabolite (sulfonium ion) which subsequently is degraded to tetrahydrothiophene (THT). THT was reported to be oxidized forming THT-1-oxide that is further oxidized to sulfolane and finally 3-hydroxysulfolane. However, the underlying mechanisms for the formation of these metabolites remain poorly understood. In the present study, we performed *in vitro* and *in vivo* investigations to elucidate the involvement of flavin-containing monooxygenase-3 (FMO3) and cytochrome P450 enzymes (CYPs) in Bu metabolic pathway. Rapid clearance of THT was observed when incubated with human liver microsomes. Furthermore, among different recombinant microsomal enzymes, the highest intrinsic clearance for THT was obtained via FMO3 followed by several CYPs including 2B6, 2C8, 2C9, 2C19, 2E1 and 3A4. In Bu- or THT-treated mice, inhibition of FMO3 by phenylthiourea significantly suppressed the clearance of both Bu and THT. Moreover, the simultaneous administration of a high dose of THT (200μmol/kg) to Bu-treated mice reduced the clearance of Bu. Consistently, in patients undergoing HSCT, repeated administration of Bu resulted in a significant up-regulation of *FMO3* and glutathione-S-transfrase -1 (*GSTA1*) genes. Finally, in a Bu-treated patient, additional treatment with voriconazole (an antimycotic drug known as an FMO3-substrate) significantly altered the Bu clearance. In conclusion, we demonstrate for the first time that FMO3 along with CYPs contribute a major part in busulphan metabolic pathway and certainly can affect its kinetics. The present results have high clinical impact. Furthermore, these findings might be important for reducing the treatment-related toxicity of Bu, through avoiding interaction with other concomitant used drugs during conditioning and hence improving the clinical outcomes of HSCT.

## Introduction

Hematopoietic stem cell transplantation (HSCT) is a curative treatment for several malignant and non-malignant disorders including leukemias, aplastic anemia, thalassemia and inborn errors of metabolism. A conditioning regimen is an important treatment prior to HSCT and consists of cytostatics with or without combination with total body irradiation (TBI). Cytostatics are non-selective drugs which eradicate malignant cells as well as normal cells [1]. In addition, conditioning contributes to elimination of the malignant cells, provides a free space for the donor cells and suppresses the host immune system in order to facilitate engraftment and to avoid graft rejection.

Busulphan (Bu) is an alkylating agent administered at high doses prior to HSCT. It reacts with DNA to form intra-strand crosslinks via the guanine and adenine nucleotide base pairs. DNA crosslinking causes permanent cell damage and triggers apoptosis.

**Fig 1:**
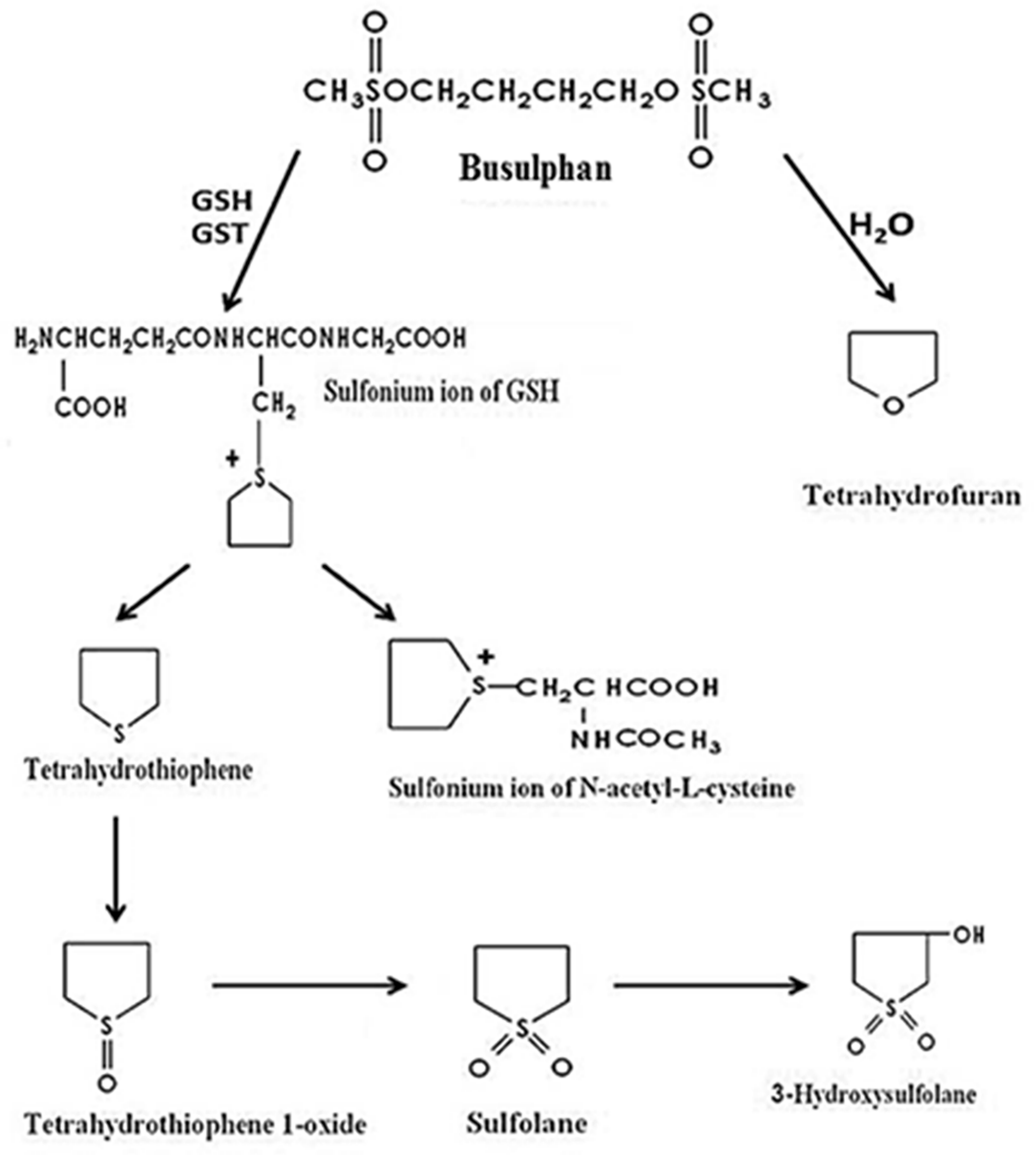
Metabolic pathway of busulphan.

Bu is metabolized mainly in the liver by conjugation with glutathione (GSH) via glutathione transferases (GSTs) [2]. This conjugation produces an unstable sulfonium ion (Fig 1) which is rapidly degraded to tetrahydrothiophene (THT). THT is the first stable metabolite of Bu that is detected in urine and plasma of rats and humans. THT is normally reabsorbed from bile and oxidized to tetrahydrothiophene 1-oxide (THT 1-oxide) which is further oxidized to sulfolane (tetrahydrothiophene 1,1-dioxide), that is finally hydroxylated to 3-hydroxysulfolane (3-OH sulfolane) [3,4].

It has been reported that only 2% of Bu is excreted in the urine as unchanged drug in rats. Furthermore, more than 70% is excreted, within 72 hours of administration, in the form of its subsequent metabolites: THT 1-oxide (20%), sulfolane (13%) and 3-OH-sulfolane (39%) [3]. Although it is well established that GSTs are involved in Bu metabolism and the formation of THT [2], scant information is available about the underlying metabolic pathways that are responsible for converting THT to THT 1-oxide, sulfolane and finally 3-OH-sulfolane. In this connection, concomitant administration of drugs such as itraconazole, metronidazole, phenytoin and ketobemidone were reported to increase Bu plasma concentrations during the conditioning therapy [5–8]. Since most of these drugs are metabolized by non-GST enzymes, these findings suggest that other enzymes are also involved in Bu metabolism. Among these enzymes, microsomal enzymes, in particular flavin-containing monooxygenases (FMO) are of high interest as they are involved in the hepatic metabolism of xenobiotics [9–11]. FMOs are mainly responsible for the metabolism of compounds containing nitrogen, sulphur and phosphorus. Five active human forms have been identified, FMO1-FMO5 [12,13]. The most common enzyme in human adult liver is FMO3 and a mutation leads to a genetic disease, trimethylaminuria [13,14].

Several attempts to incubate Bu with microsomes containing several enzymes *in vitro* have been performed, but no formation of Bu metabolites has been observed [15]. The absence of Bu metabolites could be explained by the fact that these studies employed only Bu to elucidate the role of microsomal enzymes in the formation of final Bu metabolites [15].

The present study is designed to examine the hypothesis that FMO3 and/or cytochrome P450 (CYP) enzymes are involved in the further metabolism of Bu metabolites and responsible for oxidation of THT to THT 1-oxide. We performed *in vitro*, *in vivo* mice experiments and analyzed patient samples in order to confirm our hypothesis. Our results clearly demonstrate that indeed, these enzymes contribute to the formation final Bu metabolites.

## Material and methods

### Microsomal assay

THT (Sigma-Aldrich, Stockholm, Sweden) was received as a liquid solution and stock solutions were prepared daily and diluted in 50mM potassium phosphate buffer pH 7.4.

Linearity of the reactions with time, enzyme and THT concentrations was determined before measuring apparent enzyme kinetic constants; pooled human liver microsomes (HLM) (Cypex, Dundee, UK) were incubated at different enzyme concentrations with a range of THT concentrations (10–500μM).

Time curves were performed by incubating microsomes in 50mM potassium phosphate buffer (pH 7.4) in a total volume of 200μL. The reaction was started by the addition of NADPH (Sigma-Aldrich, St. Louis, USA) to a final concentration of 1mM and terminated by adding one volume of ice cold dicholormethane (Fluka, Seeze, Germany).

The relative contribution of CYPs and FMO3 in the metabolism of THT was studied by heat-inactivation of FMO3 in some incubations [16] and, in other incubations, CYP enzymes in HLM were inactivated [17] by carbon monoxide (AGA Gas, Enköping, Sweden) bubbling.

To identify the enzymes involved in Bu metabolism, 11 microsomal batches (BD Biosciences, Stockholm, Sweden), each containing a separate cDNA-expressed enzyme, were incubated with 25μM THT. The microsomes contained FMO3, CYP1A1, CYP1A2, CYP2B6, CYP2C8, CYP2C9, CYP2C19, CYP2D6, CYP2E1, CYP3A4 or CYP4A11. Time curves (0, 5, 15, 30 and 60min) were performed at a concentration of 0.35mg protein /mL for FMO3 and 35pmol CYP/mL for the CYP enzymes. The recombinant enzymes were produced from human cDNA expressing each of them using baculovirus expression system. Baculovirus infected insect cells were used to prepare these microsomes.

Incubations were performed in triplicate and negative controls (excluding NADPH, microsomes, THT or by terminating the incubations before the addition of NADPH) were run in parallel.

To measure THT and the secondary metabolite, sulfolane, the reactions were terminated by adding dichloromethane. Samples (180μL) were added to 10μM nicotine (20μL, Merck, Hoherbrunn, Germany), used as an internal standard, before extraction. The matrix was extracted by liquid-liquid extraction with dichloromethane (equal volumes, 200μL) after 30 sec of high speed vortexing. After extraction and centrifugation at 16000 *g* for 10 min, the organic phase was transferred into GC-MS tubes. For both THT 1-oxide and 3-OH sulfolane quantification, 200μL acetonitrile (can, Merck, Darmstadt, Germany) was used to terminate the incubation. Samples (180μL) were added to 10 μM of 3-methylsulfolane (20μL, TCI, Tokyo, Japan), used as an internal standard. The matrix was lyophilized (< 1 mbar at 40°C under N_2_ stream) to dryness, the residue was dissolved in 10 μL water and 200 μL of ethyl acetate was added for extraction. Samples were vortexed for 30 sec at high speed and centrifuged at 16000 *g* for 10 min. The organic phase was transferred to GC-MS tubes.

Concentrations of THT and its metabolites were measured using GC-MS [18] after validation for use with microsomal incubations according to the international guidelines [19,20].

### Mouse study

Male C57BL/6N mice (6–8 weeks old) were purchased from Charles River (Cologne, Germany). The mice were allowed to acclimatize to the surroundings for 2 weeks with a 12h dark/12h light cycle and randomized for treatment. Standard laboratory chow and water ad libitum was introduced. Studies in animals have been carried out in accordance with the Guidelines for the Care and Use of Laboratory Animals as adopted and promulgated by the Swedish law of animal welfare. All experiments were approved by the animal ethical committee at the Stockholm South Animal Research Review Board (S119-12).

To study Bu and THT kinetics, Bu (Sigma-Aldrich, Steinheim, Germany) was administered i.p. (25mg/kg or 100μmol/kg) as a 2mM solution in DMSO (Sigma-Aldrich, Stockholm, Sweden) [21]. THT was also prepared as a 2mM solution in DMSO and the dose given was equimolar to the Bu treatment (i.e. 100μmol/kg or 8.8 mg/kg). Upon injection, samples were diluted with physiological NaCl solution to a final DMSO concentration of 10% in the dosing solution. At the appropriate time points (10min, 30min, 1h, 2h, 4h, 6h and 8h), mice (three mice at each time point) were anesthetized using 2% isoflurane and the blood samples were collected by heart puncture. Mice were euthanized by cervical dislocation. Plasma was separated and stored at -20°C.

A similar experiment was performed in a second group of mice, after 3 days of i.p. injections with the FMO3-inhibitor, Phenylthiourea (PTU, Sigma-Aldrich, Stockholm, Sweden, S1 Fig) [22]. PTU (3mg/kg) was dissolved in DMSO and diluted with physiological NaCl solution and then injected into the mice once daily followed by Bu or THT injections on the 4^th^ day. The mice were euthanized and plasma samples prepared as in the previous experiment. PTU was selected as an FMO3 inhibitor since the inhibition profile for both mouse and human FMO3 is similar [23].

To detect the effect of high THT concentrations on Bu metabolism, a third group of mice was injected with both Bu (100μmol/kg) and a high dose of THT (17.6mg/kg or 200μmol/kg) and samples prepared as described previously. All experiments including negative controls (mice injected with 10% DMSO in NaCl solution) and mice injected with PTU alone were performed in triplicates.

To quantify Bu, samples were extracted according to the method reported previously by Hassan *et al*. [24]. Briefly, 500 μL of plasma were diluted with 500 μL of water. Fifty microliters of the internal standard (1,5-bis(methanesulfonoxy)pentane; 38.5 μM) were added, followed by the addition of 1 mL of sodium iodide (8M, Merck, Darmstadt, Germany) and 400μL of *n*-heptane (Merck, Darmstadt, Germany). The tubes were placed under magnetic stirring in a water bath (70°C) for 45 min. During the reaction, busulphan and 1,5-bis(methanesulfonoxy)pentane were converted to 1,4-diiodobutane and 1,5-diiodopentane respectively and subsequently extracted with *n*-heptane [25]. The conversion of busulphan and 1,5-bis(methanesulfonoxy)pentane to 1,4-diiodobutane and 1,5-diiodopentane was over 90% [24]. The organic phase was analyzed by GC-MS [18]. THT was quantified as described previously and the method was validated for use with mouse plasma before measuring both Bu and THT [19,20].

### Study in patients

FMO3 mRNA expression was studied in twelve patients undergoing HSCT at the Center for Allogeneic Stem Cell Transplantation (CAST), Karolinska University Hospital-Huddinge, Sweden. The study was approved by the ethical committee of Karolinska Institutet (616/03) in accordance with the Helsinki Declaration of 1975 and with informed consent from each patient or their guardian. All patients were treated with oral Bu, administered at a dose of 2mg/kg (8μmol/kg) b.i.d. for 4 days followed by an i.v. infusion of cyclophosphamide (Cy) 60mg/kg/day (230μmol/kg/day) once daily for 2 days before the stem cell infusion. Patients’ clinical data are presented in Table 1.

**Table 1:** Patients’ characteristics and clinical data.

	Diagnosis	Age, (years)	Conditioning regimen	Stem cells source	Donor	CD 34 dose/Kg	Disease status, at HSCT	Acute GVHD	Outcome	Cause of death
P 1	AML	47	Bu + Cy + ATG	PBSC	MUD	8,1 x10(6)	CR1	Grade I (skin)	Alive	N/A
P 2	CML	57	Bu + Cy + ATG	PBSC	MUD	15,3 x10(6)	CP	Grade I (skin)	Alive	N/A
P 3	AML	2	Bu + Cy + ATG	BM	MUD	4,85 x10(6)	CR1	Grade I (skin)	Alive	N/A
P 4	Thalassemia Major	13	Bu + Cy + ATG	BM	HLA-id sibling	8,05 x10(6)	N/A	None	Alive	N/A
P 5	CML	14	Bu + Cy	BM	HLA-id sibling	4,8 x10(6)	CP1	Grade I (skin)	Alive	N/A
P 6	MDS-AML	50	Bu + Cy + ATG	PBSC	MUD	9,36 x10(6)	CR1	Grade I (skin)	Alive	N/A
P 7	Sickel cell anemia	13	Bu + Cy	BM	HLA-id sibling	3,39 x10(6)	N/A	Grade II (gut)	Alive	N/A
P 8	AML	35	Bu + Cy + ATG	PBSC	MUD	8,75 x10(6)	CR1	Grade I (skin)	Alive	N/A
P 9	CML	55	Bu + Cy + ATG	BM	MUD	8,19 x10(6)	CP2	Grade I (skin) + Grade III (gut)	Died, 2006-08-22	Multi organ failure
P 10	Kostmann + MDS	12	Bu + Cy + ATG	BM	MUD	4,48 x10(6)	N/A	Grade II (skin)	Died, 2006-09-22	Sudden Death
P 11	AML	54	Bu + Cy	PBSC (x3)	HLA-id sibling	3,7 x10(6)	CR1	Grade I (skin)	Alive	N/A
P 12	MDS	14	Bu + Cy + Mel + ATG	PBSC	MUD	7,9 x10(6)	PR	Grade II (skin)	Alive	N/A

**Abbreviations:** P, patient; AML, acute myeloid leukemia; CML, chronic myeloid leukemia; MDS, myelodysplastic syndrome; Bu, busulphan; Cy, cyclophosphamide; Mel, melphalan; ATG, antithymocyte globulin; PBSC, peripheral blood stem cells; BM, bone marrow; MUD, matched unrelated donor; HLA, human leukocyte antigen; CD 34, bone marrow-derived stem cells; HSCT, hematopoietic stem cell transplantation; CP, chronic phase; CR, complete remission; PR, partial remission; GVHD, graft-versus-host disease.

#### Blood sampling, RNA extraction and cDNA preparation

Blood samples for mRNA quantification were collected in PAXgene^TM^ blood RNA tubes (BD, Stockholm, Sweden) prior to the start of Bu treatment and after the last Bu dose.

RNA was extracted from the mononuclear cells using QuickPreps^TM^ Total RNA Extraction Kit (GE Life Sciences, Uppsala, Sweden). RNA (1μg) was reverse transcribed to complementary DNA (cDNA) using the TaqMan^TM^ Kit (Applied Biosystems, Roche, NJ, USA).

#### Gene array and PCR

Purified mRNA was subjected to analysis of global gene expression by using NimbleGen microarrays (Roche Diagnostics Scandinavia, Bromma, Sweden). Data were analyzed by Genespring GX (Agilent, CA, USA). Quantile normalization was used on the probe expression data generated using the Robust Multichip Average algorithm. Genes were determined to be significantly differentially expressed by ANOVA if the selection threshold of a false discovery rate was <5% and the fold change in the significance analysis of microarrays output result was >1.3.

Real-time PCR TaqMan gene expression assay (Applied Biosystems, Stockholm, Sweden) was performed by means of FAM dye labeling system. The assay was performed for FMO3 (assay ID; Hs00199368_m1) and normalized against the housekeeping-gene GAPDH (assay ID; Hs02758991_g1) (Applied Biosystems, Stockholm, Sweden).

### Clinical application

In order to confirm the contribution of FMO3 to human Bu kinetics, blood samples were collected (routinely) from a patient who underwent stem cell transplantation and was conditioned according to the hospital transplantation protocol i.e. high dose of Bu (2mg/kg b.i.d.) for four days followed with two days of cyclophosphamide (60mg/kg). The patient was treated concomitantly with voriconazole (6mg/kg, S2 Fig). Voriconazole was administered, according to the hospital clinical protocol, for the treatment of an acute fungal infection.

Samples were taken for therapeutic drug monitoring (TDM) at several time points after the first, third and fifth doses. Blood was collected into EDTA vacutainer tubes, plasma was separated by centrifugation at 1200 *g* and stored at -20°C until analysis of Bu and THT [18].

### Data and statistical analysis

Kinetic parameters from *in vivo* studies were estimated using WinNonLin software (standard edition, version 2.0), while Microsoft Excel and SigmaPlot software (version 12.5, Systat software, Inc.) were utilized for *in vitro* kinetics. Graphs were plotted using GraphPad Prism software (version 4.0, GraphPad Software, Inc.)

The data and statistical analysis complied with the recommendations on experimental design and analysis in pharmacology and the level of probability (P) for t-test is deemed to constitute the threshold for statistical significance when *P*<0.05.

## Results

### Involvement of microsomal enzymes in THT metabolism

Incubation of THT with HLM showed rapid THT disappearance. Furthermore, inactivation of FMO3 in the microsomes (heat treatment) reduced the THT disappearance by 44% within 60 min. On the other hand, CYP inhibition by carbon monoxide resulted in the reduction of THT disappearance by only 9%. Thus, THT is metabolized by microsomal enzymes with FMO3 as the main enzyme, but with contribution also from CYPs.

**Fig 2:**
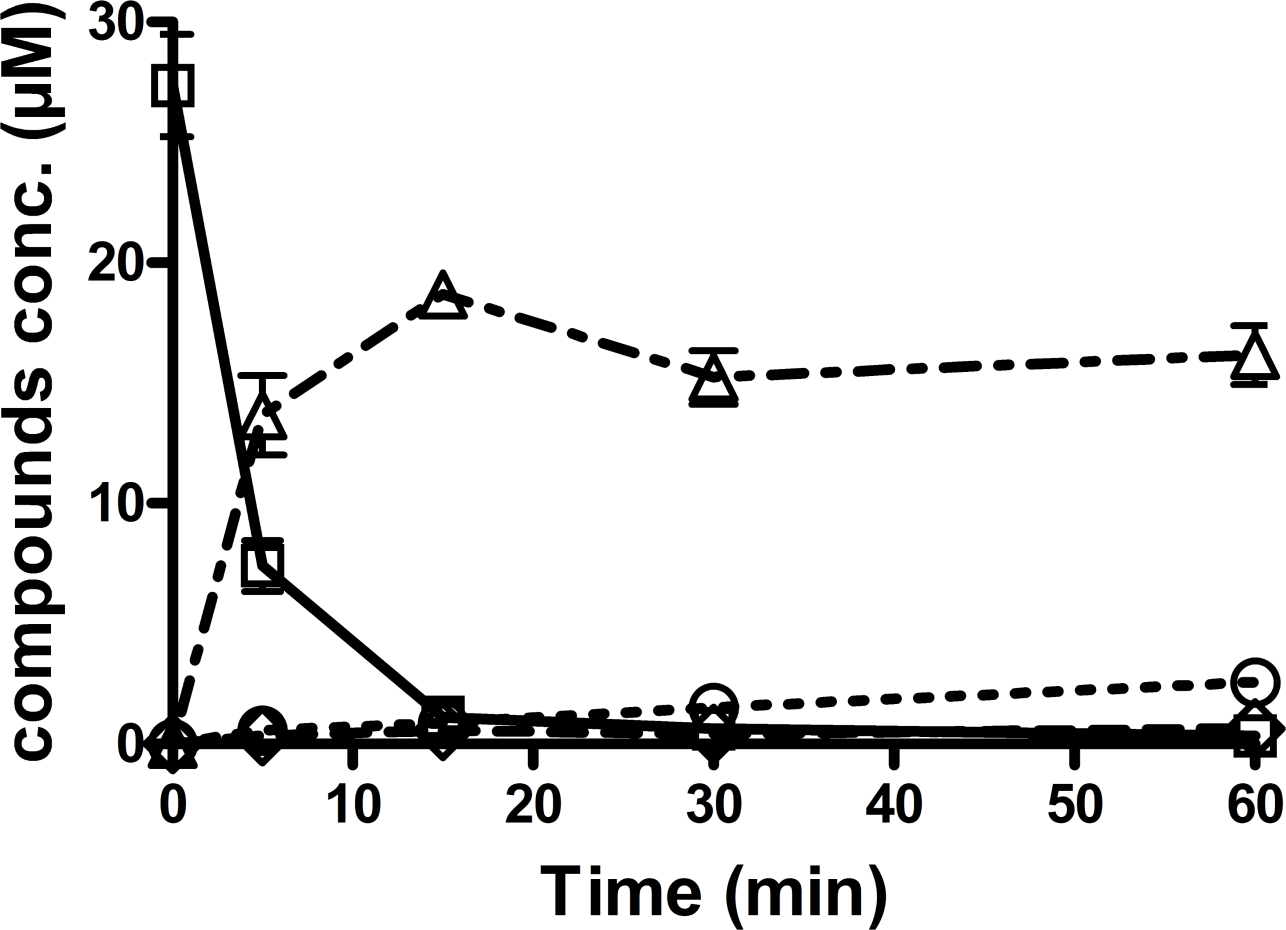
Concentration-time curves for tetrahydrothiophene and its three metabolites after incubation with recombinant FMO3. Microsomes with cDNA-expressed human FMO3 were incubated with 25μM tetrahydrothiophene (THT). A time curve (0, 5, 15, 30 and 60min) was performed with triplicate incubations and a protein concentration of 0.35mg/mL. 96% of THT was metabolized by recombinant FMO3 in 15 min. The disappearance was accompanied by the formation of metabolites, mainly THT 1-oxide. The other two metabolites (sulfolane and 3-OH-sulfolane) started to appear at later time points, in line with sequential metabolism. (□) THT, solid line; (Δ) THT 1-oxide, dash/dotted; (○) sulfolane, dotted; (◊) 3-OH-sulfolane, solid line.

To further confirm the involvement of FMO3 in Bu metabolism, the disappearance of THT was measured by incubation of this compound with the recombinant FMO3. As shown in Fig 2, THT disappeared very rapidly i.e., 96% of this Bu metabolite disappeared within 15min, with a formation of subsequent metabolites, THT 1-oxide, sulfolane and 3-OH-sulfolane (Fig 2). Although with great variation, recombinant CYPs in particular, CYP2B6, CYP2C8, CYP2C9, CYP2C19, CYP2D6, CYP3A4 and CYP4A11 also induced THT disappearance (not shown).

With respect to the involvement of microsomal enzymes in Bu metabolism, incubations with recombinant FMO3 exerted the highest initial THT disappearance rate (*v*_*0*_) (6.87μmol/min/mL) followed by CYPs 3A4, 4A11, 2C8 and 2D6 (Table 2). FMO3 also yielded the highest intrinsic clearance (*CL*_*int*_ value, 709.14μL/min/mg protein) followed by CYPs 3A4, 2C9 and 2C8 (Table 2).

**Table 2:** Apparent enzyme kinetics for tetrahydrothiophene disappearance by recombinant human enzymes.

Enzyme	*CL*_*int*_ value (μL/min/mg protein)	*CL*_*int*_ value (μL/min/pmol CYP)	*v*_*0*_ (nmol/min/mL)	*v*_*0*_/CYP (nmol/min/pmol CYP)
FMO3	709.14	—	6.87	—
CYP1A1	84.70	1.36	1.68	0.05
CYP1A2	108.20	0.91	1.24	0.04
CYP2B6	276.02	1.85	1.76	0.05
CYP2C8	470.96	1.93	2.45	0.07
CYP2C9	632.55	1.45	1.64	0.05
CYP2C19	437.70	1.36	1.51	0.04
CYP2D6	220.95	2.05	2.24	0.06
CYP2E1	78.89	1.38	1.41	0.04
CYP3A4	697.02	3.35	3.66	0.11
CYP4A11	186.61	2.43	2.52	0.07

Microsomes with cDNA-expressed enzymes were incubated with tetrahydrothiophene (THT, 25μM). A time curve (up to 60min) was performed, where FMO3-containing microsomes were incubated at a protein concentration of 0.35mg/mL and CYP-containing microsomes were incubated at 35pmol CYP/mL. Incubations were performed in triplicate and started with the addition of NADPH. FMO3 had the highest CL_int_ value and initial THT disappearance rate (v_0_) followed by several CYPs.

### Inhibition of FMO3 activity suppresses the clearance of busulphan in mice

**Fig 3:**
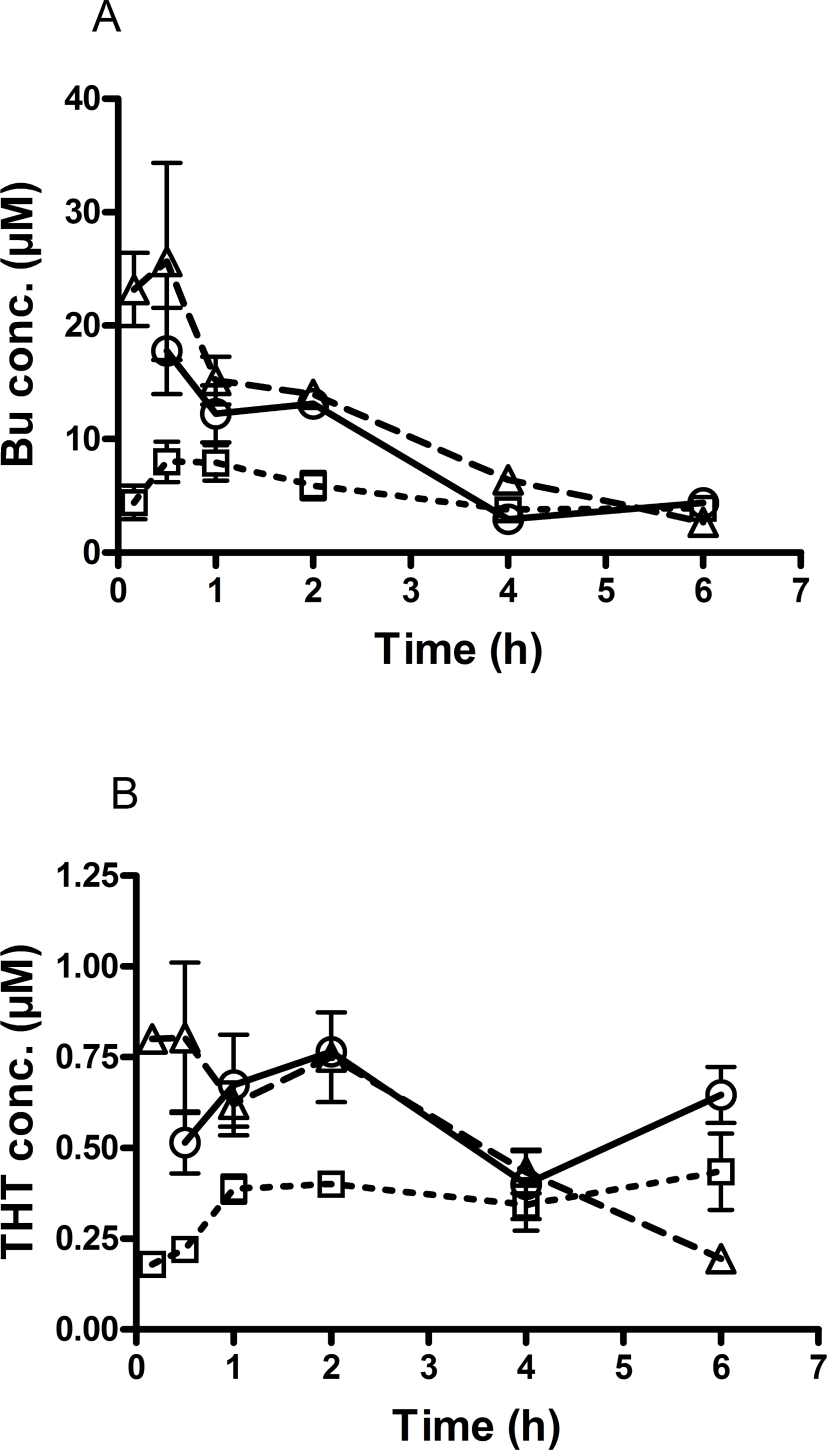
The effect of FMO3 inhibition on concentration-time curve of busulphan (Bu) and tetrahydrothiophene (THT) in mice. To study the effect of FMO3 inhibition on Bu kinetics, animals were divided into three groups. All experiments were run with 3 mice per time point in each group. Animals were euthanized and blood samples collected at different time points. Group1. A single dose of Bu (25mg/kg) was administered i.p. to mice Group2. A single dose of Bu (25mg/kg) was administered i.p. 24h after pretreatment with the FMO3-inhibitor phenylthiourea (PTU, 3mg/kg; i.p.) for 3 days. Group3. Bu (25mg/kg) was administered concomitantly with a high dose of THT (17.6mg/kg). **A.** Pretreatment with PTU significantly (P<0.05) increased plasma levels of Bu (Δ, dashed line) compared to that observed when Bu was administered alone (□, dotted line). THT, when given together with Bu, also affected Bu metabolism and increased in Bu plasma concentrations (○; solid line) compared to Bu alone. **B.** Pretreatment with PTU significantly (P<0.05) increased plasma concentrations of Bu first stable metabolite (THT) in mice injected with Bu (Δ, dashed line) compared to that observed when Bu was administered alone (□, dotted line). THT concentrations in mice injected with Bu and PTU (Δ, dashed line) were almost the same as in mice injected with Bu and concomitant high doses of THT without pretreatment with PTU (○; solid line).

Our above findings that FMO3 plays a pivotal role in the clearance of the Bu metabolite THT *in vitro*, led us to evaluate whether this enzyme also affects the metabolism of Bu *in vivo*. As shown in Fig 3 and Table 3, Bu plasma concentrations and Bu exposure expressed as area under the concentration-time curve (AUC) were significantly (*P*<0.05, t-test) higher in mice treated with the FMO3 inhibitor, PTU before Bu administration as compared to the mice administered with Bu alone.

**Table 3:** Busulphan kinetics in mice.

	Group 1		Group 2		Group 3	
	Mean	SEM	Mean	SEM	Mean	SEM
**AUC (μM.h)**	44.29	5.79	65.33	1.27	65.51	8.57
**K01 (h)**	0.39	0.19	0.08	0.01	0.16	0.08
**K10 (h)**	3.07	1.31	1.67	0.38	2.32	0.12
**CL (μM.h)**	40.73	8.00	30.64	0.60	31.64	4.27
**C_max_ (μM)**	7.47	0.98	25.54	6.26	16.51	2.72

Busulphan (Bu) was injected i.p. (25mg/kg) into mice. At appropriate time points (10min– 8h), mice were euthanized and blood samples collected (Group 1). The same experiment was performed after 3 days of i.p. injection of the FMO3-inhibitor phenylthiourea (PTU). PTU (3mg/kg) was injected once daily followed by Bu injection on the 4^th^ day and plasma samples collected as for Group 1 (Group 2). To detect the effect of high concentrations of tetrahydrothiophene (THT) on Bu metabolism, a third group of mice was injected with both Bu and a high dose of THT and sampled as above (group 3).

PTU injection increased Bu AUC and reduced its clearance. High THT concentrations due to concomitant administration of THT and Bu also affected Bu kinetics in a similar manner as after an injection of PTU.

Furthermore, THT concentrations were also significantly (*P*<0.05, t-test) increased in mice injected with Bu and PTU compared to those injected with Bu alone (Fig 3B). Concomitant administration of THT and Bu also resulted in higher levels of Bu compared to those received Bu alone (Fig 3A and Table 3).

THT concentrations in mice injected with Bu and PTU were almost at the same level as in mice injected with Bu plus a double dose of THT (Fig 3B).

**Fig 4:**
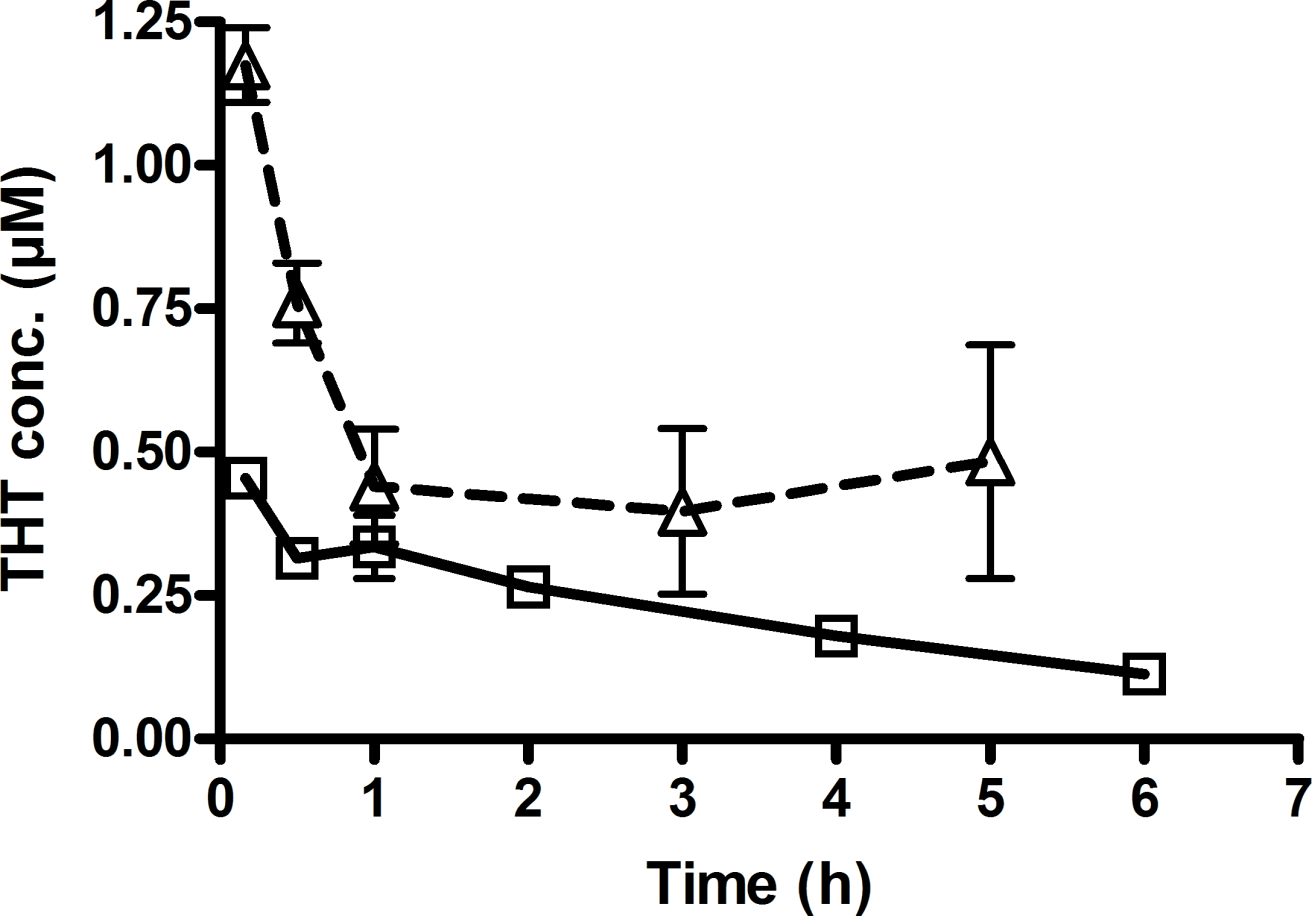
The effect of FMO3 inhibition on concentration-time curve of tetrahydrothiophene (THT) in mice. To study the effect of FMO3 inhibition on THT kinetics, animals were divided into two groups. All experiments were run with 3 mice per time point in each group. Animals were euthanized and blood samples collected at different time points. Group 1. A single dose of THT (8.8mg/kg) was administered i.p. to mice. Group 2. THT (8.8mg/kg) was administered 24h after pretreatment with the FMO3-inhibitor phenylthiourea (PTU, 3mg/kg; i.p.) for 3 days. Pretreatment with PTU significantly (P<0.05) increased plasma levels of THT (Δ, dashed line) compared to that observed when THT was administered alone (□, solid line).

THT concentrations were also significantly (*P*<0.05, t-test) higher in THT-treated mice pretreated with PTU compared to mice receiving THT only (Fig 4).

### FMO3 gene expression in patients conditioned with busulphan prior to HSCT

**Fig 5:**
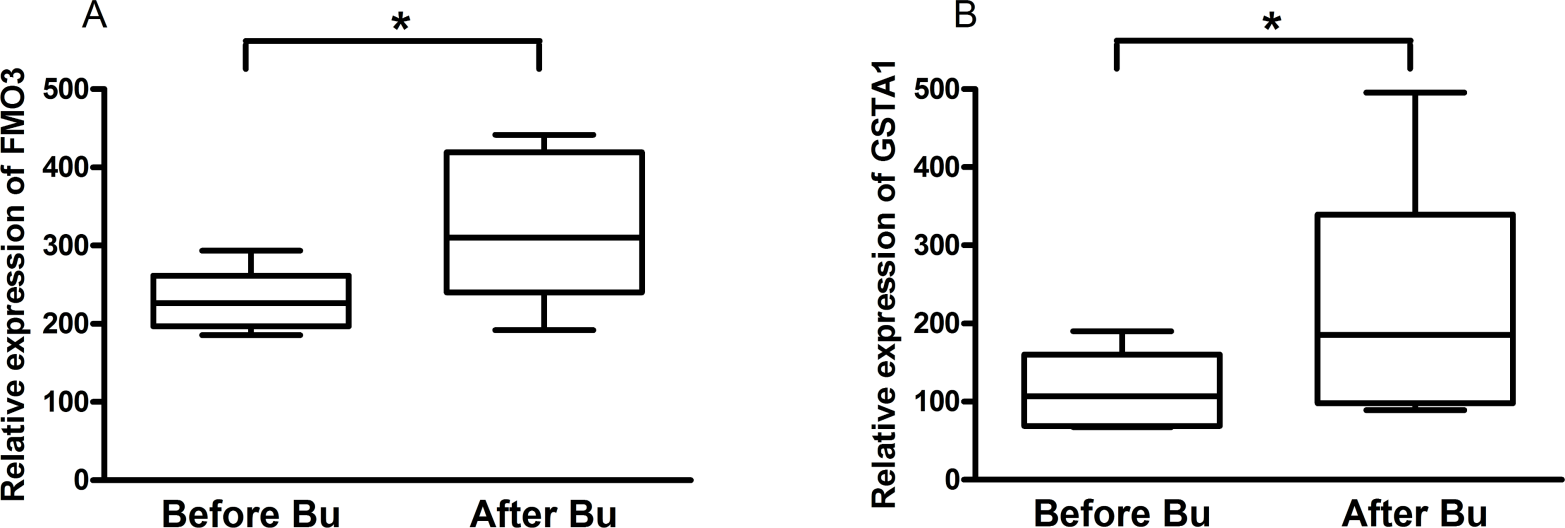
**The effect of busulphan treatment on mRNA of FMO3 (A) and GSTA1 (B) in patients during conditioning prior to HSCT.** *The gene array analysis showed that FMO3 gene expression was significantly (P<0*.*05) increased after busulphan (Bu) conditioning (2mg/kg b*.*i*.*d*. *for 4 days) compared to before the start of Bu conditioning (confirmed by qRT-PCR)*. *The expression was normalized to that of GAPDH*. *The inter-individual variation in the relative expression increased from 2*.*8-fold before Bu conditioning*, *to 5*.*6-fold by the end of the treatment*. *GSTA1 was also significantly up-regulated (P<0*.*05)*. *Inter-individual variation in the relative expression of GSTA1 increased from 1*.*6-fold before Bu conditioning to 2*.*3-fold by the end of the treatment*.

In order to elucidate the role FMO3 in Bu metabolism in human, we measured the expression of *FMO3* gene in patients treated with Bu prior to HSCT. A significant up-regulation (*P*<0.05, t-test) of mRNA was found for *FMO3* after Bu conditioning. *FMO3* up-regulation followed the same pattern as that observed for *GSTA1* expression (*P*<0.05, t-test) (Fig 5). *FMO3* up-regulation was confirmed by real time PCR for *FMO3* gene expression normalized against *GAPDH*. The inter-individual variation in the relative expression of *FMO3* increased from 2.8-fold before Bu conditioning to 5.6-fold by the end of the treatment and, for *GSTA1*, the variation increased from 1.6-fold before Bu conditioning to 2.3-fold by the end of the treatment.

No correlation between clinical data (diagnosis, type of donor, stem cell dose, relapse, remission, mortality and complications) and the *FMO3* expression was observed (Table 1).

### Interference of FMO3 substrate, voriconazole with busulphan metabolism

**Fig 6:**
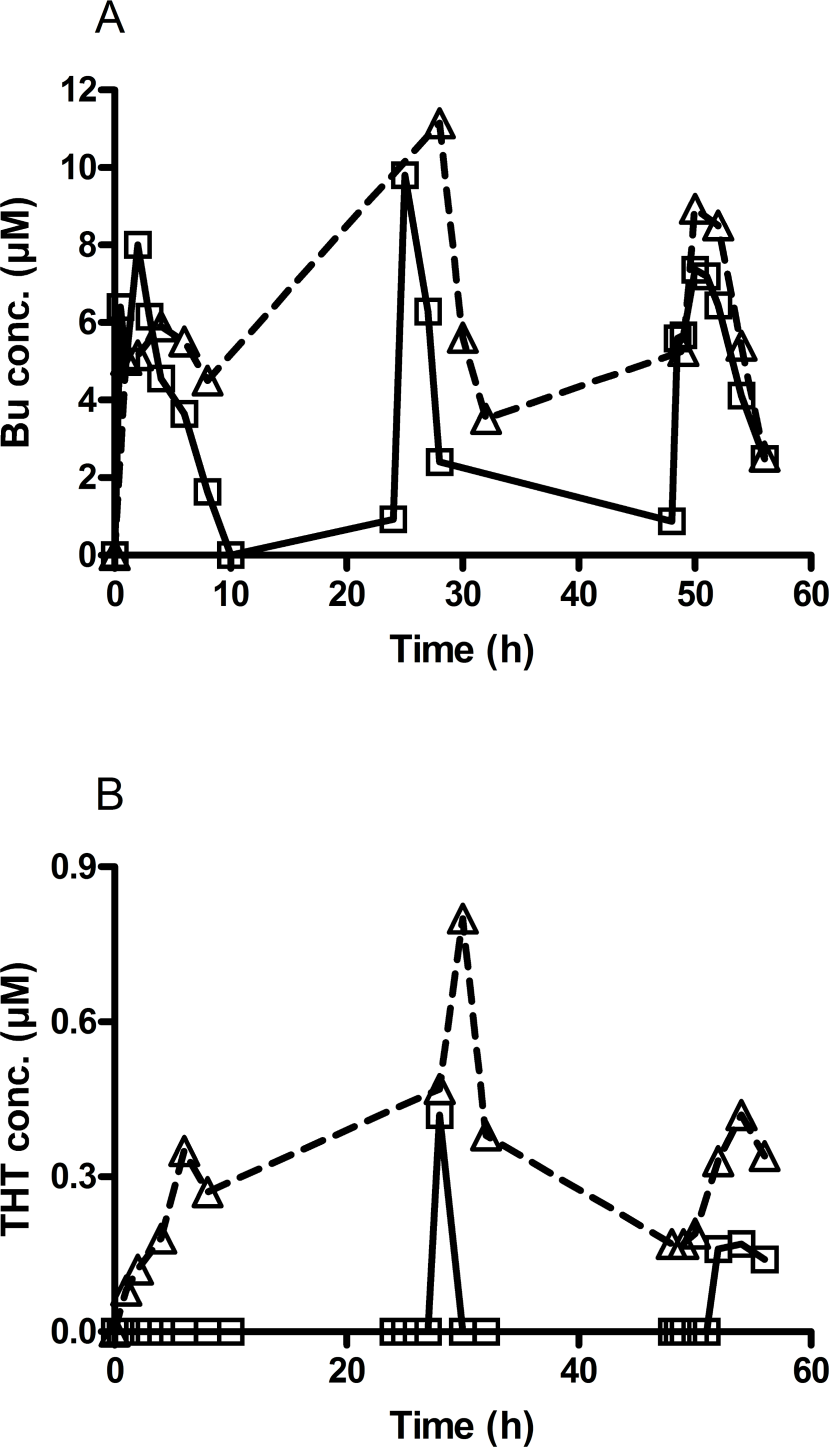
The effect of voriconazole treatment on busulphan and tetrahydrothiophene kinetics in patients conditioned prior to HSCT. Busulphan (Bu) and tetrahydrothiophene (THT) were determined in two patients undergoing stem cell transplantation and conditioned with high doses of busulphan (2mg/kg b.i.d.) for four days. One patient received only Bu during conditioning, while the other patient was concomitantly administered voriconazole for Candida infection (3mg/kg i.v. b.i.d). Samples were collected at several time points for therapeutic drug monitoring. (A) Significantly (P<0.05) higher levels of Bu were observed in the patient receiving concomitant voriconazole (Δ, dashed line), compared to the levels observed after the same dose of Bu when administered alone (□, solid line). (B) THT levels were significantly (P<0.05) higher already after the first Bu dose with high accumulation and slower metabolism (Δ, dashed line) compared to Bu alone (□, solid line). Voriconazole was withdrawn after Bu second dose and Bu dose was reduced to 1.2mg/kg b.i.d for two days.

Finally, our above results led us to investigate whether those drugs that are known to act as substrate for FMO3 would interfere with the metabolism of Bu. As shown in Fig 6A, Bu levels were higher than expected in a patient treated concomitantly with Bu and voriconazole, compared to the levels seen after the same dose of Bu administered alone. THT was detected at 1h after the first dose of Bu and reached higher concentrations than with Bu alone (Fig 6B). Bu clearance was also slower compared to a patient treated with Bu alone (*P<*0.05, t-test) [18]. Voriconazole was withdrawn after Bu second dose and the Bu dose was reduced to 1.2mg/kg b.i.d for two days.

## Discussion

In the present study, for the first time, we provide evidences that FMO3 and CYPs are involved in the final stages of Bu metabolism. As a highly lipophilic compound, Bu is first metabolized to its more hyrdrophilic metabolites (sulfonium ion) via conjugation with GSTs, in particular GSTA1 [26]. GSTA1 polymorphisms and other isoforms, such as GSTP1 and GSTM1, have been reported to affect Bu kinetics [26–28]. The primary unstable GSH-conjugate is degraded to produce THT. Unfortunately, THT is lipophilic compound that has to be reabsorbed and oxidized to be further excreted.

In this study, we have shown that both FMO3 and, to less extent, CYPs are responsible for THT oxidation to THT 1-oxide, also may play a role in the downstream oxidation of THT 1-oxide. In this connection, incubating THT with HLM resulted in a rapid disappearance of THT followed by the formation of secondary Bu metabolites. THT 1-oxide was the primary metabolite formed, while both sulfolane and 3-OH sulfolane appeared later. The findings that heat-inactivation of FMO3 in the microsomes reduced THT disappearance by 44%, whereas CYP inhibition by carbon monoxide reduced THT disappearance by only 9% clearly suggest that FMO3 contributes more than the CYPs in down-stream metabolism of THT. In fact, this suggestion was strongly supported by our studies on THT incubations with recombinant enzymes. For instance, FMO3 showed the highest initial THT disappearance rate and also the highest *CL*_*int*_ value followed by several CYPs such as 2B6, 2C8, 2C9, 2C19, 2D6, 3A4 and 4A11. In line with this latter observation, previous studies have also shown a role for CYP2C9 in the overall Bu metabolism [29].

Although FMO3 seems to be the main enzyme for THT oxidation, several CYPs are also involved in this process. The disappearance of THT is shown to be a fast reaction which means that the electron transfer from cytochrome P450 oxidoreductase (POR) to CYP may be the rate limiting step [30]. POR is the main electron donor for all microsomal CYP monooxygenases and its polymorphism has been shown to affect CYP-mediated drug metabolism such as CYP2B6 and CYP2C9 [31–34]. The variability in POR activity may contribute to the inter individual variation known for busulphan kinetics [2,4,5,26–28].

FMO3 is the major FMO isoform expressed in the liver of adults. In embryos, only low levels of FMO3 are present. By the age of two, FMO3 is detectable in most individuals and is expressed at intermediate levels until 11 years of age [35]. FMO1 is expressed mainly in the fetus and embryo, and decreases within 3 days postpartum [36]. Bu kinetics is known to be age dependent [37–40], possibly due to age-dependent FMO3 expression. Until 2005, none of the FMO3 polymorphisms were reported to be associated with an adverse drug reaction [35].

Consistent with our *in vitro* results, our *in vivo* findings showed that inhibition of FMO3 with the selective inhibitor, PTU induced THT accumulation in Bu-treated mice. Moreover, in mice dosed with THT, THT concentrations were also significantly higher after PTU injection, compared to mice injected with THT alone. These results clearly indicate that FMO3 also plays a pivotal role in the metabolism of THT *in vivo*.

The accumulation of THT during Bu administration also resulted in a significant decrease in Bu clearance, possibly by feedback inhibition (i.e. an enzyme in a biosynthetic pathway may be inhibited by an end product of that pathway). Co-administration of THT concomitantly with Bu, also significantly increased Bu concentrations and AUCs compared to that found in mice injected with Bu alone.

We have reported previously an interaction between Bu and metronidazole in patients undergoing HSCT [6]. The concomitant treatment resulted in two-fold higher mean Bu trough levels; sadly THT quantitative method was not available at that time. Another case study of a 7-year-old boy with AML reported decreased Bu clearance by 46% after two days concomitant treatment with metronidazole. It was shown that the daily AUC significantly exceeded (by 86%) the predicted as a result of drug-drug interactions. Metronidazole administration was instantly stopped after observing this drug interaction [41].

Similar results were also observed in a 24-year old female, where Bu levels increased after starting concomitant treatment with fluconazole [42]. Other drugs with FMO3- or CYP-dependent metabolism, such as itraconazole and ketobemidone, also lead to higher plasma concentrations of Bu [5,7].

In patients undergoing HSCT, *FMO3* gene expression showed a marked up-regulation of mRNA by the end of Bu-conditioning after the 4^th^ day. Likewise, *GSTA1* was up-regulated after Bu-conditioning. The *GSTA1* and *FMO3* expression levels, as well as gene polymorphism, may have clinical impact on the personalized treatment of busulphan.

During the current investigation, a patient undergoing HSCT was treated with Bu and concomitant voriconazole (for the treatment of fungal infection). Bu levels were significantly higher than expected for the administered dose. Voriconazole was withdrawn after the first dose of Bu and, subsequently, Bu concentrations decreased to the expected levels. Moreover, early appearance of THT in the plasma, higher accumulation and slower clearance of THT were also seen compared to what has previously been reported when Bu was administered alone [18]. Yanni *et al*. have reported that 25% of voriconazole is metabolized via FMO3 [43]. Voriconazole could thus be a competitive inhibitor of FMO3-dependent THT metabolism.

Several studies have reported that FMO3 is involved in the metabolism of many drugs including voriconazole, ketoconazole, methimazole, tamoxifen, codeine and nicotine [43–45]. Some of these drugs are co-administered during conditioning with Bu as prophylactic treatment during transplantation, which may explain the high Bu exposures reported in those patients. Besides the GST involvement, the acquired knowledge about the role of FMO3 and CYPs is of a great importance for patients where polypharmacy is common.

Cyclophosphamide (Cy) is an alkylating agent administered after Bu as a part of the conditioning regimen. Cy is a prodrug that is bioactivated mainly by CYP2B6 and several other CYPs such as CYP3A4 and CYP2C9 [26,46,47]. In this study, we showed that several CYPs are capable of metabolizing THT. Many of Cy end-metabolites are toxic [48] and any Bu metabolites affecting the activity of enzymes involved in Cy bioactivation may also alter Cy-efficacy and hence its toxicity. We have previously reported that the time interval between Bu and Cy affect Cy-kinetics and hence the incidence of liver toxicity [49]. Moreover, reversing the sequence of administration of Bu/Cy, i.e. administrating Cy before Bu, was reported to reduce the hepatotoxicity both in patients and mice [50,51]. Recently, several groups have shown that sinusoidal obstruction syndrome (SOS) and liver function tests in addition to the day +100 mortality were significantly reduced in patients conditioned with Cy followed by Bu [52,53]. However, further investigations are required to address the effect of THT on CYP activities and their effect on Cy metabolism.

Based on the discussion above, Bu/Cy conditioning protocols should be revised to take the drug administration sequence, and/or time interval between both drugs, into account in addition to considering the concomitant supportive therapy.

## Conclusion

**T**his is the first report showing that FMO3, in addition to CYPs, is involved in the metabolism of THT, the first stable metabolite of Bu, and that FMO3 can affect the overall Bu clearance. FMO3 inhibition affects Bu and THT kinetics *in vivo* in mice and humans. Drugs metabolized by FMO3 and/or CYPs, and used concomitantly during Bu conditioning, will significantly alter busulphan kinetics and hence its exposure, treatment efficacy and toxicity. Further studies are warranted to address the effect of gene polymorphisms and expression levels of metabolizing enzymes on busulphan metabolism and kinetics. The present results can be utilized for personalized medicine which in turn may affect the treatment related toxicity and hence improve the clinical outcome in HSCT patients.

## Supporting information

S1 Fig Chemical structure of Phenylthiourea (PTU). (TIF)

S2 Fig Chemical structure of voriconazole. (TIF)
