# Correction: Flavin-containing monooxygenase 3 (FMO3) role in busulphan metabolic pathway

**DOI:** 10.1371/journal.pone.0190181

**Published:** 2017-12-19

**Authors:** The PLOS ONE Staff

The following information is missing from the Funding section: This study was supported by a Radiumhemmets research grant (161082).
