# Supplementary figures and images for: Flavin-containing monooxygenase 3 (FMO3) role in busulphan metabolic pathway
Source: PLoS One. 2017 Nov 9;12(11):e0187294. doi: 10.1371/journal.pone.0187294 (PMC5679629; doi:10.1371/journal.pone.0187294)

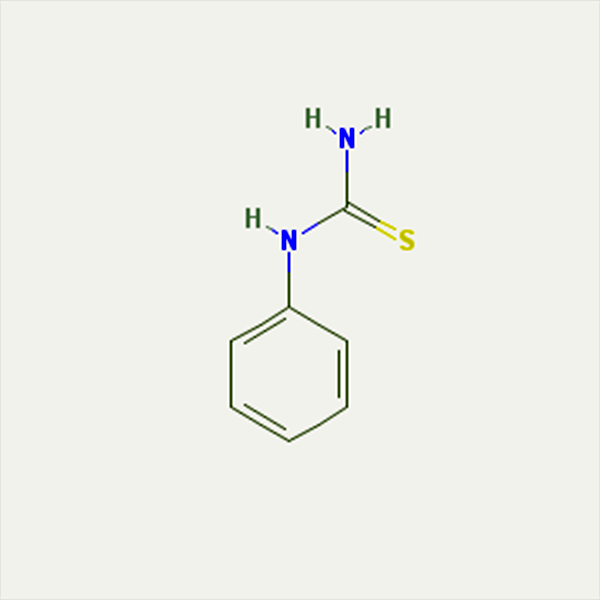

Supplement: S1 Fig — (TIF) [file pone.0187294.s001.tif]

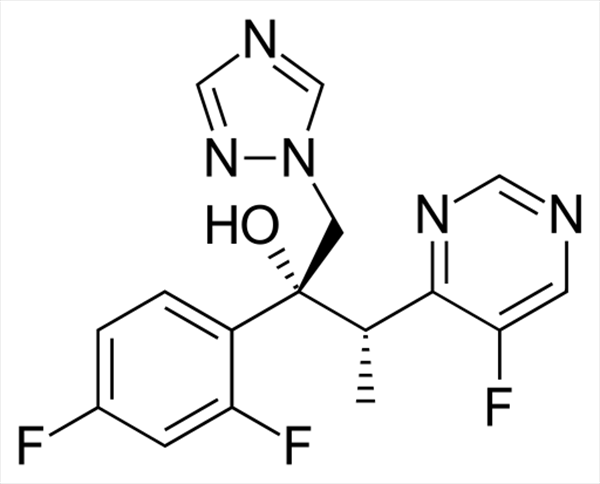

Supplement: S2 Fig — (TIF) [file pone.0187294.s002.tif]
